# Automatic segmentation and quantification of the optic nerve on MRI using a 3D U-Net

**DOI:** 10.1117/1.JMI.10.3.034501

**Published:** 2023-05-15

**Authors:** Sabien van Elst, Christiaan M. de Bloeme, Samantha Noteboom, Marcus C. de Jong, Annette C. Moll, Sophia Göricke, Pim de Graaf, Matthan W. A. Caan

**Affiliations:** a Amsterdam UMC location Vrije Universiteit Amsterdam, Department of Radiology and Nuclear Medicine, Amsterdam, The Netherlands; b Cancer Center Amsterdam, Imaging and Biomarkers, Amsterdam, The Netherlands; c Amsterdam UMC location Vrije Universiteit Amsterdam, Department of Anatomy and Neurosciences, Amsterdam, The Netherlands; d Amsterdam UMC location Vrije Universiteit Amsterdam, Department of Ophthalmology, Amsterdam, The Netherlands; e University Hospital Essen, Institute of Diagnostic and Interventional Radiology and Neuroradiology, Essen, Germany; f Amsterdam UMC location University of Amsterdam, Department of Biomedical Engineering and Physics, Amsterdam, The Netherlands

**Keywords:** optic nerve, segmentation, quantification, U-Net, magnetic resonance imaging

## Abstract

**Purpose:**

Pathological conditions associated with the optic nerve (ON) can cause structural changes in the nerve. Quantifying these changes could provide further understanding of disease mechanisms. We aim to develop a framework that automatically segments the ON separately from its surrounding cerebrospinal fluid (CSF) on magnetic resonance imaging (MRI) and quantifies the diameter and cross-sectional area along the entire length of the nerve.

**Approach:**

Multicenter data were obtained from retinoblastoma referral centers, providing a heterogeneous dataset of 40 high-resolution 3D T2-weighted MRI scans with manual ground truth delineations of both ONs. A 3D U-Net was used for ON segmentation, and performance was assessed in a tenfold cross-validation (n=32) and on a separate test-set (n=8) by measuring spatial, volumetric, and distance agreement with manual ground truths. Segmentations were used to quantify diameter and cross-sectional area along the length of the ON, using centerline extraction of tubular 3D surface models. Absolute agreement between automated and manual measurements was assessed by the intraclass correlation coefficient (ICC).

**Results:**

The segmentation network achieved high performance, with a mean Dice similarity coefficient score of 0.84, median Hausdorff distance of 0.64 mm, and ICC of 0.95 on the test-set. The quantification method obtained acceptable correspondence to manual reference measurements with mean ICC values of 0.76 for the diameter and 0.71 for the cross-sectional area. Compared with other methods, our method precisely identifies the ON from surrounding CSF and accurately estimates its diameter along the nerve’s centerline.

**Conclusions:**

Our automated framework provides an objective method for ON assessment *in vivo*.

## 1 Introduction

The optic nerves (ONs) play a vital role in visual perception by connecting the retina to the brain. Morphologically, the ONs are thin, tortuous structures that bend from the eye globe to the optic chiasm and exhibit shape variations as well as variable amounts of surrounding cerebrospinal fluid (CSF) along their pathway.^1^^,^^2^ Pathological conditions, such as multiple sclerosis (MS), optic neuritis, ON hypoplasia, and optic pathway gliomas can cause damage to the ON that coincides with impaired vision.^3^ Furthermore, intraocular tumors such as retinoblastoma can invade the ON, which is a poor prognostic indicator and a risk factor for metastatic disease.^4^ Pathologies affecting the ON can lead to structural changes such as ON atrophy or enlargement that locally affect its diameter and volume.^5^^–^^9^ Hence, accurate quantification of the ON’s diameter along the length of the nerve can provide valuable insights into the nature and progression of various pathologies, as well as assist in treatment planning and monitoring.

Magnetic resonance imaging (MRI) is an effective imaging modality for visualizing the ONs and detecting pathological changes due to its high soft-tissue contrast and the avoidance of ionizing radiation.^10^ Advances in MRI techniques have enabled the acquisition of high-resolution 3D T2-weighted MR images that, unlike computed tomography (CT) and T1-weighted MRI, produce a distinct contrast between the ON and its surrounding CSF.^11^ However, segmenting and quantifying the ONs is challenged by their complex morphology.^12^ Manual annotation by an experienced radiologist is therefore time-consuming, labor-intensive, and prone to inter- and intrarater variability, emphasizing the need for automatic methods for accurate ON segmentation and quantification.

In recent years, automatic approaches for ON segmentation on MRI have been developed. These methods typically focus on jointly segmenting the ON and surrounding CSF. Atlas-based approaches apply registration to an anatomical reference for segmentation. Panda et al.^13^ presented a multiatlas labeling approach for segmenting the eyes, ONs with surrounding CSF, and optic chiasm from high-resolution 3D T2-weighted MR images. Crouzen et al.^14^ developed an MR-based organs-at-risk (OARs) segmentation atlas from cerebral T1-weighted MR scans to automatically delineate OARs, including the ONs, for radiotherapy planning. However, their ON segmentations required frequent manual adjustments to achieve clinical acceptability. Statistical shape models incorporate the expected appearance and shape information of an object, constraining its segmentation to anatomically plausible shapes. A fully automated partitioned statistical shape model (ASM) for anterior visual pathway segmentation in both healthy and abnormal subjects was presented by Mansoor et al.^15^ Their method involved extensive preprocessing, including feature engineering on multisequence MRI data. Nguyen et al.^16^ proposed an ASM for automatically segmenting intraocular structures, including part of the ONs, in patients with uveal melanoma on T1-weighted MRI, obtaining accurate segmentation of the most distal part of the ONs only. Although atlas-based approaches and statistical shape models produce anatomically consistent results, they are generally limited by poor generalization capacity, which especially affects ON segmentation due to the large variability in shape and appearance.^13^^,^^16^

Deep learning models have been proposed to advance in various medical imaging tasks, including improving our clinical understanding of the ON.^17^ Automatic segmentation of the ON using deep learning has been explored in several studies, such as for studying glaucoma progression^18^^,^^19^ or diagnosing increased intracranial pressure.^20^^,^^21^ On MRI, deep learning techniques for automatic ON segmentation have primarily been investigated in the context of OARs segmentation for radiotherapy planning, where they have been shown to outperform atlas-based approaches.^22^ Liu et al.^23^ implemented a 3D U-Net architecture combined with a cycle-GAN to synthesize MR images from CT images for segmenting OARs. They demonstrated that synthetic MR images provide complementary information and improved segmentation performance compared with conventional automatic CT segmentation. A 2D U-Net was used by Mlynarski et al.^24^ to segment multiple OARs including the ON on contrast-enhanced T1-weighted MRI scans. Dai et al.^25^ recently developed a regional convolutional neural network for segmenting OARs on MRI, incorporating several advanced architectures, such as a deep attention feature pyramid network and mask scoring networks, but their ON segmentation performance was low compared with other organs included in the study. Li et al.^26^ proposed a parallel stages network composed of two 2D U-Nets for segmentation of the entire anterior visual pathway, employing T1-weighted MR images and fractional anisotropy images for feature extraction. They targeted the entire anterior visual pathway and did not separate both ONs and chiasm.^26^

To accurately quantify the ON and its disease-related changes, it is necessary to differentiate the ON from the surrounding CSF rather than segmenting them as a single structure. Few studies have aimed at isolating the ON from surrounding CSF to obtain precise quantitative measures of the ON itself. One such study by Harrigan et al.^27^ used a multiatlas segmentation method to initially segment the ON with CSF on T2-weighted MR images. They then employed a model based on the difference between Gaussians to fit the intensity values of the ON and CSF in the coronal plane, allowing for independent measurements of the ON and CSF diameter along the length of the nerve. However, this model may not hold for high-resolution imaging, where variable thickness of the CSF layer is expected. Another study by Feng et al.^28^ proposed a segmentation method using gradient-based edge detection with skeletonization to delineate the ON without the CSF on coronal 3D T1-weighted MRI. Their method required prior knowledge of ON location assigned by the user. Previous methods extracted the cross-sectional area and diameter of the ON in the coronal plane, which may result in an overestimation of the actual size due to the tortuous structure of the ON that is not always aligned perpendicular to the coronal plane.^12^

To overcome the limitations of previous methods, we propose a 3D pipeline for automatic segmentation and quantification of the ON, while accurately differentiating it from CSF along the entire length of the nerve. Our pipeline consists of two main steps. First, we employ a 3D U-Net to automatically segment the ON from high-resolution T2-weighted MR images obtained from multiple centers to allow for precise segmentation of the ON without CSF. Second, we implement a quantification method that uses the resulting 3D segmentations to extract ON diameter and cross-sectional area along the centerline of the nerve. This automatic approach addresses the limitations of previous quantification methods by enabling quantitative measurements independent of image intensity values or ON orientation. Our study demonstrates the feasibility and effectiveness of this pipeline for automatic segmentation and quantification of the ON, which can serve as a valuable tool for accurately characterizing the ON and its disease-related changes on MRI.

## 2 Methods

### 2.1 Data Acquisition

Data were collected retrospectively from two retinoblastoma referral centers in Essen, Germany, and Amsterdam, the Netherlands, as high-resolution T2-weighted MRI scans were readily available in the pediatric retinoblastoma population. The Institutional Review Board of the Amsterdam UMC, location VUmc, Amsterdam, The Netherlands, approved this multicenter retrospective study with waiver of informed consent. The dataset consisted of 40 subjects (mean age 2.83±1.76 years), with a total of 28 healthy and 52 pathology-affected eyes. MRI data were acquired on a 1.5-Tesla scanner (Magnetom Aera, Siemens, Erlangen, Germany) and a 3.0-Tesla scanner (Discovery MR750, GE Healthcare, Chicago, United States). The acquisition protocol included a high-resolution 3D T2-weighted sequence, i.e., CISS (Siemens) and FIESTA (GE). Acquisition parameters are summarized in Table 1. Scans had anisotropic voxel sizes and variable field of views, yet always included the patient’s head, both eyes, and the entire left and right ON. During MR acquisition, patients were under general anesthesia. Manual ON segmentations were performed by an experienced reader (C.M.d.B, 5 years of experience) using 3D Slicer^29^ (Version 4.10.1) and included the full length of the right and left ON reaching up to the optic chiasm. Annotations were validated by two neuroradiologists (P.d.G and M.C.d.J, 17 and 10 years of experience, respectively).

**Table 1:** MRI acquisition parameters.

	Amsterdam (n=25)	Essen (n=15)
Manufacturer	GE Medical Systems	Siemens
Sequence	FIESTA	CISS
Repetition time (ms)	8.0 [7.0 to 8.6]	13.3 [12.1 to 13.3]
Echo time (ms)	3.5 [3.4 to 3.5]	6.6 [6.1 to 6.6]
Flip angle	40 deg	80 deg
In-plane resolution (mm)	0.27 × 0.27 [0.27 to 0.29]	0.25 × 0.25 [0.25 to 0.34]
Slice thickness (mm)	0.3	0.7
Matrix (voxels)	512 × 512 × 96 [92 to 112]	360 [340 – 420] x 320 x 64 [56 to 80]
Field strength (Tesla)	3.0 T	1.5 T

Values are presented as median [min to max range].

### 2.2 Proposed Framework

**Fig. 1:**
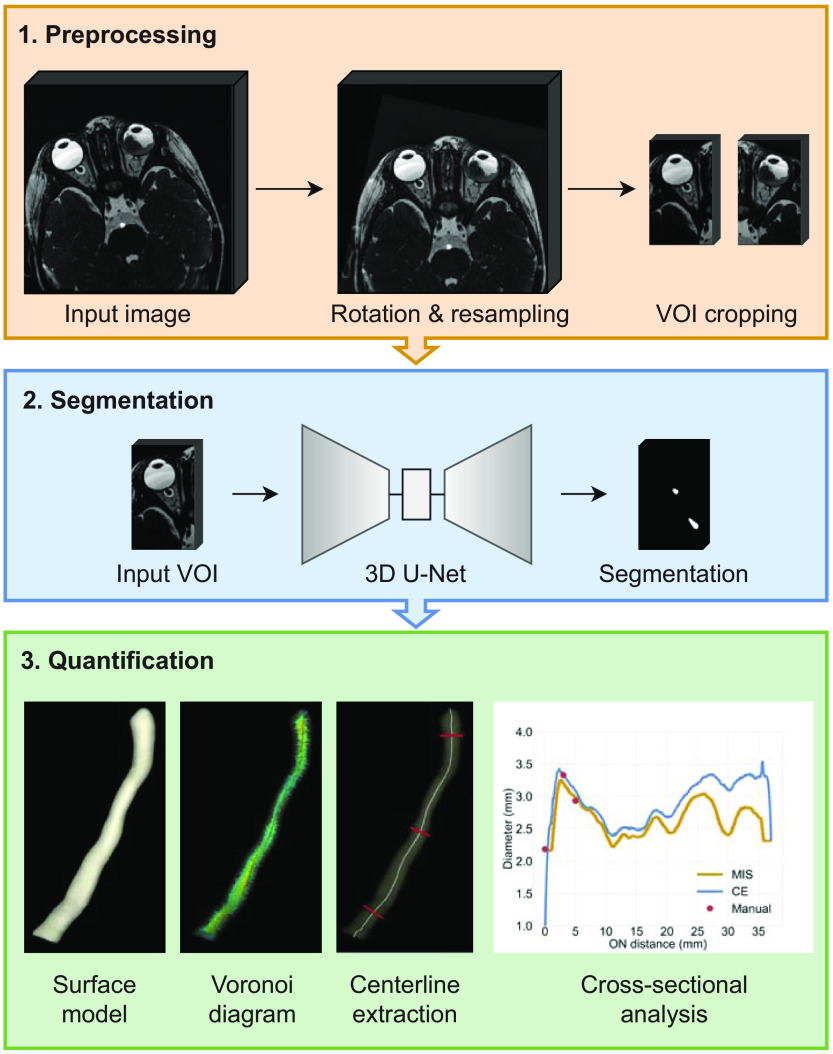
Schematic overview of the proposed framework, performing segmentation of the ON separate from surrounding CSF, and computation of the diameter profile in the cross-sectional plane along the ON.

The overall proposed framework is composed of three primary processes; (1) data preprocessing, (2) ON segmentation, and (3) ON diameter and cross-sectional area quantification. The segmentation network architecture is based on the U-Net, one of the most used and widely known architectures for medical image segmentation.^30^ The resulting segmentations are input to a quantification method, which is based on centerline extraction of tubular 3D models to enable precise cross-sectional size estimation. A comprehensive overview of the framework is shown in Fig. 1. Each process is explained in more detail in Secs. 2.2.1–2.2.3.

#### 2.2.1 Preprocessing

**Fig. 2:**
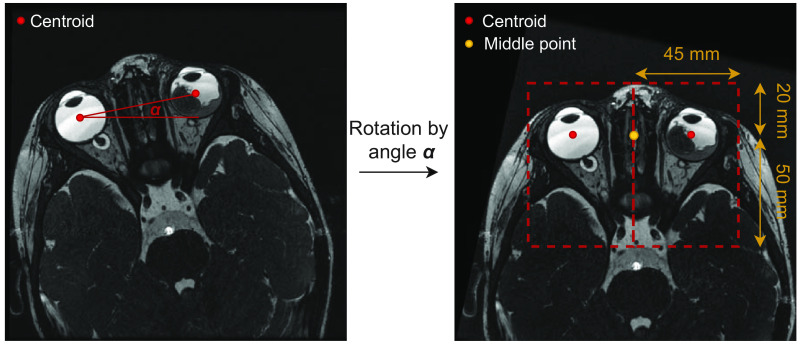
Example of scan rotation and VOI cropping. Scans are rotated by angle α based on their centroid (red dots). After rotation, scans are cropped into two VOIs using the middle-point between the centroids (orange dot). Red dotted lines represent VOI cropping area. Note: Image is 3D in reality, while the example is 2D for visualization purposes.

Since MRI data originated from multiple centers and imaging protocols, preprocessing procedures were applied to homogenize the data. Bias field correction was applied using the N4-ITK algorithm^31^ to correct for intensity nonuniformity. Due to GPU memory constraints, scans were cropped into two smaller volumes of interest (VOI), each including one eye and its corresponding ON. VOI extraction was initiated by automatic detection of the eyes’ centroids using the 3D Hough filtering approach of the Insights Toolkit.^32^ To ensure consistent VOIs of the entire ON without encompassing part of the contralateral ON, scans were rotated such that the centroids of both eyes were aligned to correct for head rotation. An example is shown in Fig. 2. The angle of rotation was determined between the left and right eyes’ centroids, and realignment of the scan was performed if the angle was greater than five degrees to avoid correcting for insignificant differences. Rotation was applied simultaneously with resampling to limit intermediate interpolation using the FMRIB Software Library^33^ (FSL). Images were resampled to an isotropic spatial resolution of $0.3\;{\mathrm{mm}}^{3}$. Subsequently, a cropping area was applied by extending the middle point between two centroids about 50 mm posteriorly, 20 mm anteriorly, 17 mm superiorly and inferiorly, and 45 mm left or right to include the right or left eye and ON, respectively, based on a representative subject (see Fig. 2). The resulting VOIs of 144×240×112  voxels were visually inspected to verify that the ON was entirely included. Lastly, voxel values within each VOI were normalized to have a mean of 0 and a variance of 1.

#### 2.2.2 Segmentation

##### Network architecture

**Fig. 3:**
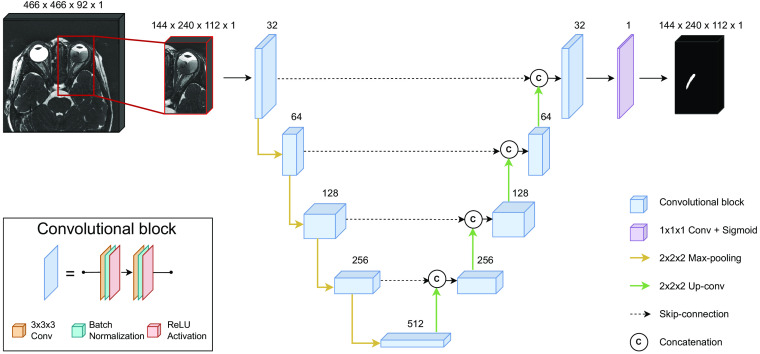
Architecture of the segmentation network. Number of feature maps is specified above each block.

A 3D U-Net was implemented as segmentation network (Fig. 3). Both the encoding and decoding part of the U-Net are assembled with five layers of convolutional blocks, in which the number of features maps is gradually changed (i.e., 32, 64, 128, 256, 512). The convolutional block encompasses two 3×3×3 convolutions, each followed by batch normalization and a rectified linear unit as activation function. In between the layers of the encoding path, input dimensions are reduced by a 2×2×2  max-pooling with strides of two to extract both spatial and context features. In the decoder path, layers are connected by an upconvolution operation of 2×2×2 with strides of two to produce full-resolution segmentations. At each layer in the decoding path, feature maps are concatenated with feature maps from layers of equal resolution in the encoding path, prior to passing through the convolutional block. These skip-connections allow the passage of low-level and high-level features from the encoding path to the decoding path and hence regain detailed information that was removed during downsampling. In the final layer, a 1×1×1 convolution followed by a sigmoid activation is applied to reduce the number of output channels to the number of labels (in our case 1: ON = 1; background = 0). Both the input and output of the network is a 144×240×112 volume with one channel. In supplementary experiments, residual units^34^ and attention gating blocks^35^ were added to the 3D U-Net architecture to evaluate their impact on model performance.

##### Model training

The dataset of each center was randomly split into two subsets for training and testing with a partition rate of 0.8 and 0.2, respectively, corresponding to a combined total of 32 subjects (i.e., 64 eyes) in the training subset and eight subjects (i.e., 16 eyes) in the testing subset. During training, a tenfold cross-validation analysis was conducted to assess the network’s performance. Following these experiments, the network was retrained on the entire training dataset and subsequently evaluated on the test-set. To improve model robustness and avoid overfitting, data augmentation was implemented. On-the-fly augmentation encompassed random scaling (0.8 to 1.2, p=0.5), left-right flipping (p=0.5), intensity shifting (0.5 to 1.5, p=0.3), additive Gaussian noise (sigma = 0.1, p=0.3), and Gaussian blurring (0.2 to 1.2, p=0.3). A Dice loss was used as loss function for its robustness to class imbalance.^36^

##### Implementation details

Our model was implemented in Python 3.6.9 using Keras 2.1.0 with TensorFlow 2.2.0 backend. Training was executed on an NVIDIA GeForce GTX 1080 TI GPU (11 GB memory) using CUDA 10.1 for accelerated training. The Adam optimizer was used for optimization and its learning rate was set to $1\times 10^{-3}$. The training batch size was restricted to one image per batch due to GPU memory restriction. Models were trained for up to 150 epochs. An early stopping strategy was utilized if there was no improvement in validation loss after 40 epochs. After each epoch, model weights were saved if an improvement in validation loss was observed. Training took ∼4  h. Inference time was a few seconds per image.

##### Evaluation metrics

For performance evaluation, several commonly used evaluation metrics for medical image segmentation were used, including Dice similarity coefficient (DSC), precision, recall, Hausdorff distance 95th percentile (HD95), average surface distance (ASD), and intraclass correlation coefficient (ICC). Spatial agreement between automatic segmentation and manual reference was measured by the DSC:

$$\mathrm{DSC}(A,B)=\frac{2(A\cap B)}{\left|A|+|B\right|},$$ (1)

where A and B refer to the set of voxels in the manual and automatic segmentations, respectively.

The Hausdorff distance (HD) and ASD are effective indicators to assess contour similarity.^37^ HD measures the largest distance between the boundary points of the manual and automatic segmentation. The 95th percentile of the HD is used to eliminate outliers:

$$\mathrm{HD}95(A,B)=\max_{95\%}(\mathrm{hd}(A,B),\;\mathrm{hd}(B,A)),$$ (2)

with

$$\mathrm{hd}(X,Y)=\underset{x\in X}{\max}\;\underset{y\in Y}{\min}\|x-y\|.$$ (3)

The ASD measures the average distance between the two surfaces and is defined as

$$\mathrm{ASD}(A,B)=\frac{1}{\left|A|+|B\right|}(\underset{a\in A}{\sum }d(a,\;B)+\underset{b\in B}{\sum }d(A,b)).$$ (4)

Volumetric agreement between the manual and automatic segmentation was quantified by the ICC (two-way mixed effects, single rater).

#### 2.2.3 Quantification

To measure the diameter and cross-sectional area along the length of each ON, segmentations produced by the segmentation network were analyzed in 3D Slicer^29^ (version 5.0.3), an open-source software for medical image computing and visualization. The workflow for ON size quantification was scripted in Slicer’s Python interpreter for automation and consists of two components: centerline extraction and size assessment. Extraction of the nerve’s centerline enables diameter measurements along the length of the nerve, regardless of its orientation to the imaging plane. First, the raw segmentation of the ON, created by the segmentation network, was loaded into Slicer as a 3D surface model. Then, the centerline of the model was determined using the Vascular Modeling Toolkit^38^ (VMTK). VMTK is an extension of Slicer that provides modules for vascular modeling, including centerline extraction and cross-sectional analysis of a 3D model.^39^ Since the tubular structure of the ON roughly resembles vascular structures, we extended its application to ON quantification. Centerline extraction was initiated by automatically identifying the model’s starting and ending points. Manual adjustments were made if automatic detection was inadequate. Between these endpoints, the centerline of the model was computed based on the Voronoi diagram, which represents the center points of the maximal inscribed spheres inside the model.^39^ The extracted centerline and surface model were adopted as input for VMTK’s cross-sectional analysis module, which measures the cross-sectional area, its circular-equivalent (CE) diameter, and the maximum inscribed sphere (MIS) diameter at a sampling distance of 0.1 mm along the model.

##### Performance evaluation

Automatic measurements of the ON’s diameter and cross-sectional area were compared with manual measurements made by experienced radiologists. Manual measurements were made on the same dataset used in this study at three measurement points: at the level of the most anteriorly located CSF (as close to the lamina cribrosa as visually possible) and at 3 and 5 mm posterior from this point. Agreement between automatic and manual measurements at these points was evaluated by the mean absolute error (MAE) and ICC score (two-way mixed effects, single rater). Since the ONs were annotated to extend into the sclera at the level of the lamina cribrosa, the origin of the ON surface models did not exactly correspond to the origin of the manual measurement points. To allow for fair comparison, the average offset between the surface model origin and the manual measurement origin was found by minimizing their MAE. Bland–Altman difference plots were created to visually analyze agreement between manual measurements and automatic measurements.

To evaluate the performance of our method and compare it to existing approaches, we conducted two analyses. First, the parameterized approach of Harrigan et al.^27^ was implemented, which segments the ON and CSF using an atlas-based registration method and fits a multivariate Gaussian model to estimate the ON diameter. Since the atlas is not publicly available, we performed a manual segmentation of the ON including CSF for one representative subject. Subsequently, we replicated their approach by fitting a Gaussian mixture model in MATLAB (MathWorks, Natick, Massachusetts) to extract the ON from the CSF. Second, we conducted an ablation study to compare the accuracy of our cross-sectional analysis method to existing methods that estimate ON diameter in the coronal plane (as discussed in Sec. 1). The cross-sectional area $a_{\mathrm{cs},i}$ and the coronal area $a_{\mathrm{cor},i}$ were computed for each location i along the centerline of the ON. Let us consider the local orientation vector of the centerline of the ON $c_{i}$ and the normal vector of the coronal plane $v_{\mathrm{cor}}$. Now, $a_{\mathrm{cor},i}$ is expected to overestimate $a_{\mathrm{cs},i}$ dependent on the local orientation $c_{i}$ relative to $v_{\mathrm{cor}}$. To demonstrate this, we compute a corrected coronal area through $a_{\mathrm{cor},i}$ ($c_{i}{\cdot v}_{\mathrm{cor}}$) and an estimated angle of the centerline through $\arccos(c_{i}{\cdot v}_{\mathrm{cor}})$. We then computed the relative error in the estimated equivalent ON diameter as a function of the arc length of the centerline and averaged it over all test subjects.

## 3 Results

### 3.1 Segmentation

**Fig. 4:**
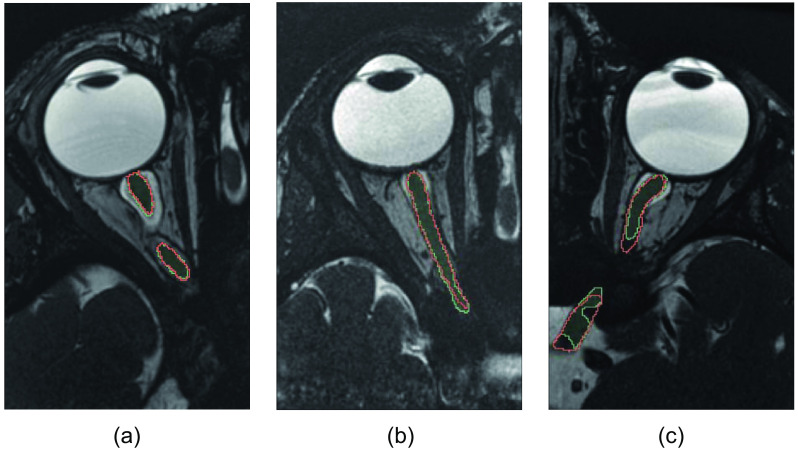
Visual comparison of segmentation results: (a) high performance (DSC = 0.90), (b) average performance (DSC = 0.83), and (c) low performance (DSC = 0.75). Each image illustrates the segmentation on a single axial slice. Green denotes the segmentation produced by our method, and red denotes the manual ground truth.

Examples of segmentation results obtained by our method, as compared with manual segmentation, are shown in Fig. 4. Each image illustrates the performance of the method on a single axial slice. For a more comprehensive visualization of the segmentation performance in 3D, please refer to Fig. S1 in the Supplementary Materials, which provides one segmentation example across all slices. The average spatial agreement achieved by our model was a DSC of 0.83±0.04 for the tenfold cross-validation and a similar DSC of 0.84±0.04 on the test-set (Table 2). By allowing a margin of one voxel tolerance to account for uncertainty at the borders of the manual segmentation, the DSC was increased to 0.90 and 0.91, respectively.

**Table 2:** Quantitative segmentation performances for tenfold cross-validation and test set.

Metric	Tenfold cross-validation (n=64)	Test-set (n=16)
**Spatial (mean ± STD)**		
DSC	0.83 ± 0.04	0.84 ± 0.03
DSC (tolerance 1 voxel)	0.90 ± 0.06	0.91 ± 0.04
Precision	0.85 ± 0.05	0.84 ± 0.04
Recall	0.82 ± 0.09	0.85 ± 0.04
**Distance (median [IQR])**		
HD95	0.60 [0.42 to 0.86]	0.60 [0.42 to 1.02]
ASD	0.14 [0.10 to 0.17]	0.14 [0.12 to 0.17]
**Volumetric**		
ICC	0.88	0.95

n, number of eyes; STD, standard deviation; IQR, inter quartile range; DSC, Dice similarity coefficient; HD95, Hausdorff distance 95%; ASD, average surface distance; ICC, intraclass correlation coefficient.

**Fig. 5:**
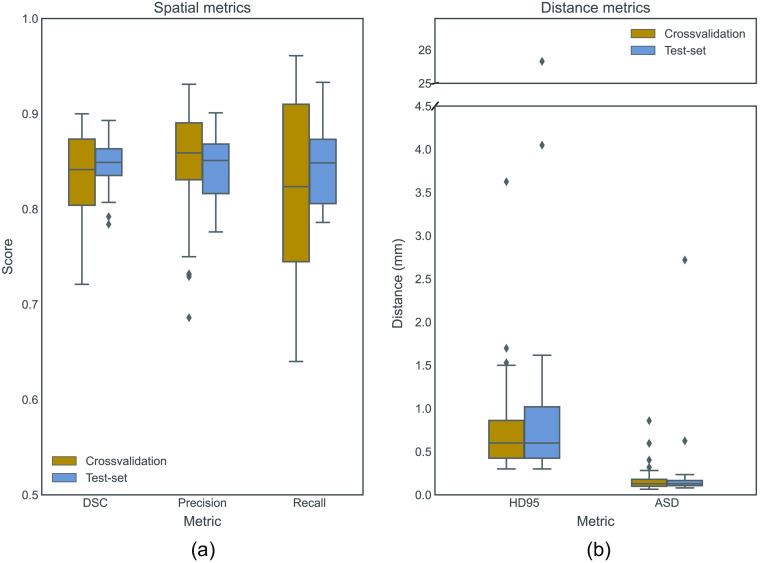
Boxplots of (a) spatial metrics and (b) distance metrics. DSC, Dice similarity coefficient; HD95, Hausdorff distance 95%; ASD, average surface distance; ICC, intraclass correlation coefficient.

Boxplots of the spatial evaluation metrics and distance metrics are shown in Fig. 5. It can be observed that the HD is affected by some extreme outliers, which were caused by few misclassified voxels far outside the ON region. These outliers can be discarded by retaining only the largest connected component, reducing the mean HD from 2.44 to 0.66 mm on the test set. ICC scores show good (0.88) to excellent (0.95) volumetric agreement between manual reference and automatic segmentation. The results of the tenfold cross-validation analysis, as shown in Table S1 in the Supplementary Materials, indicate that the expansion of the network architecture through the addition of supplementary blocks, i.e., residual units or attention gating, did not result in performance improvement.

### 3.2 Quantification

**Fig. 6:**
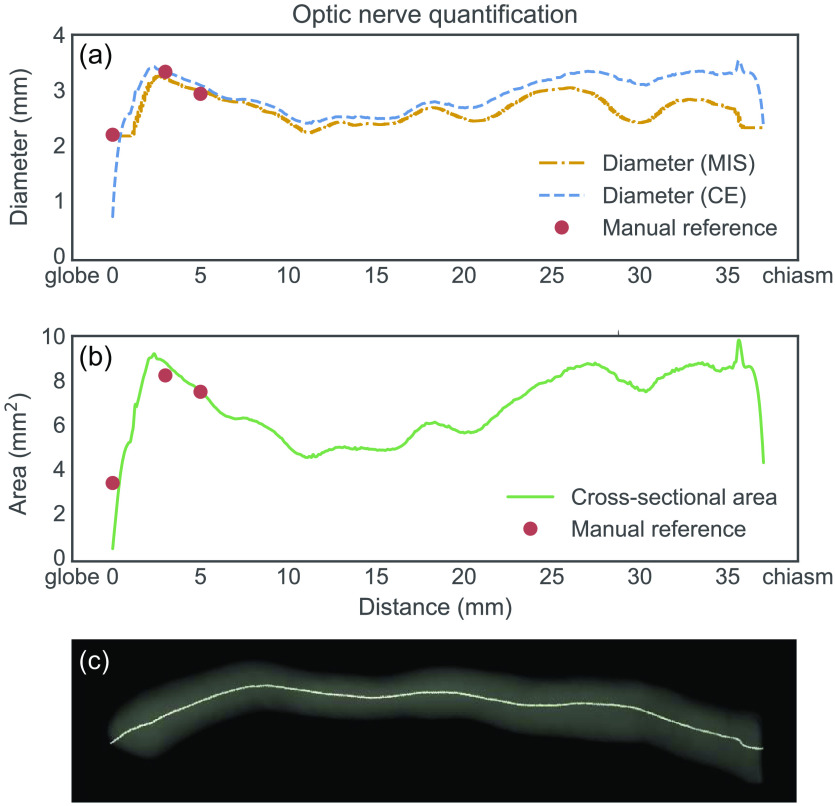
Example of (a) diameter and (b) cross-sectional area measurement of ON from eye globe to optic chiasm with manual reference measurements at 0, 3, and 5 mm. (c) The corresponding ON surface model with centerline. MIS, maximum inscribed sphere; CE, circular equivalent.

Diameter and cross-sectional area measurements were obtained from the ON segmentations. Figure 6 shows a graphic representation of the ON, displaying the diameter and cross-sectional area from eye globe to optic chiasm with corresponding manual reference measurements, as well as the corresponding 3D surface model with its centerline.

**Fig. 7:**
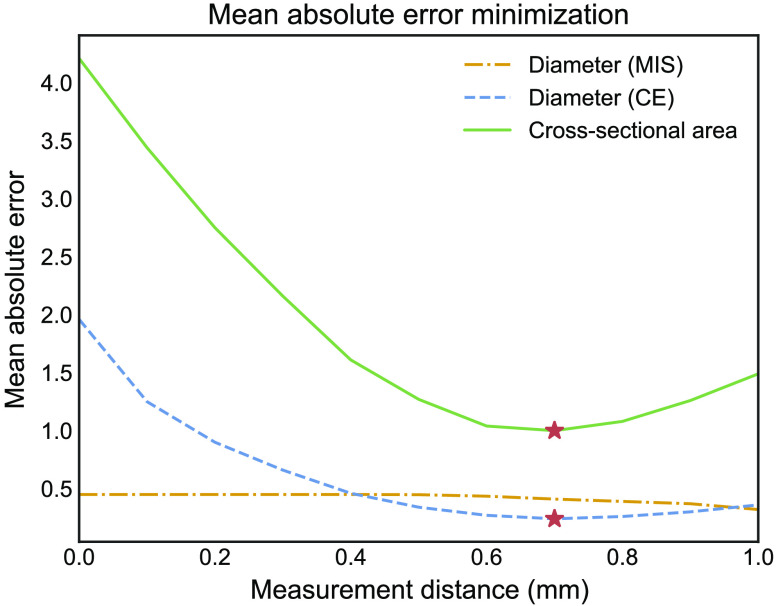
MAE minimization between manual measurement at the most anteriorly CSF and automatic measurements. Red star represents the lowest error estimation for diameter and cross-sectional area measurements, occurring at 0.7 mm distance from the model’s origin. MIS, maximum inscribed sphere; CE, circular equivalent.

**Fig. 8:**
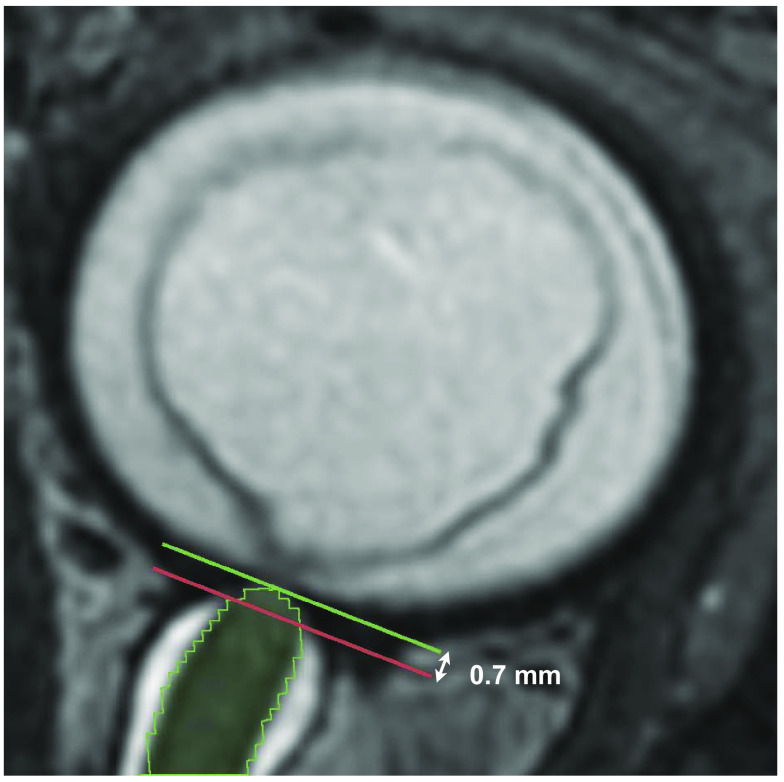
Example of manual reference measurement at the most anteriorly located CSF (red line) versus ground truth origin at globe (green line) with an offset of 0.7 mm.

As mentioned in Sec. 2.2.3, a minor offset existed between the automatic measurements and the manual reference points. By comparing the absolute error between the manual measurement at the most anteriorly located CSF and the automatic measurements between 0 and 1 mm distance of the model’s origin, an average offset of 0.7 mm was found based on MAE minimization (see Fig. 7). Figure 8 shows that this offset between the ON origin and the level of the most anteriorly located CSF is anatomically feasible. Consequently, for each subject, the three manual measurements were compared with the automatic measurements at 0.7, 3.7, and 5.7 mm. The MAE at these measurement points was 0.24±0.27, 0.22±0.24, and 0.26±0.28  mm, respectively, for the CE diameter and 0.42±0.39, 0.33±0.32, and 0.38±0.32  mm for the MIS diameter. Cross-sectional area measurements had an MAE of 1.0±1.3, 1.1±1.4, and $1.2\pm 1.4\;{\mathrm{mm}}^{2}$, respectively. Overall ICC values for CE diameter, MIS diameter, and cross-sectional area measurements were 0.76, 0.72, and 0.71, respectively.

**Fig. 9:**
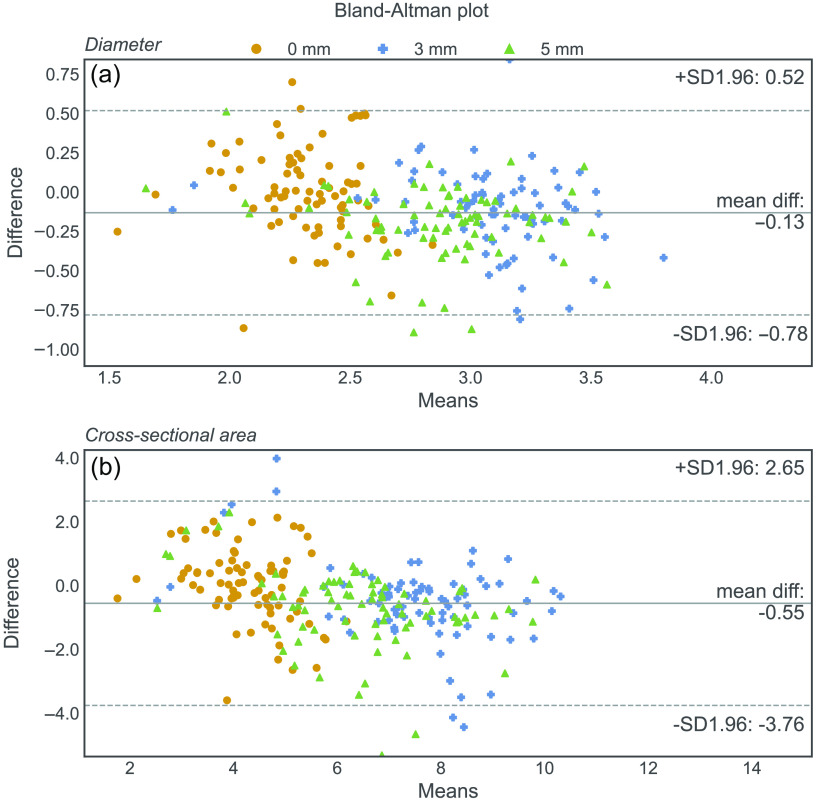
Bland–Altman difference plots of (a) diameter measurements and (b) cross-sectional area measurements.

Figure 9 shows the Bland–Altman difference plots of the automatic measurements compared with the manual measurements. The vast majority of the measurements fall within the 95% limits of agreement and their mean is approximately zero. There appears to be a slight tendency for overestimation of the smaller sizes and underestimation of the larger sizes for both the diameter and the cross-sectional area measurements.

**Fig. 10:**
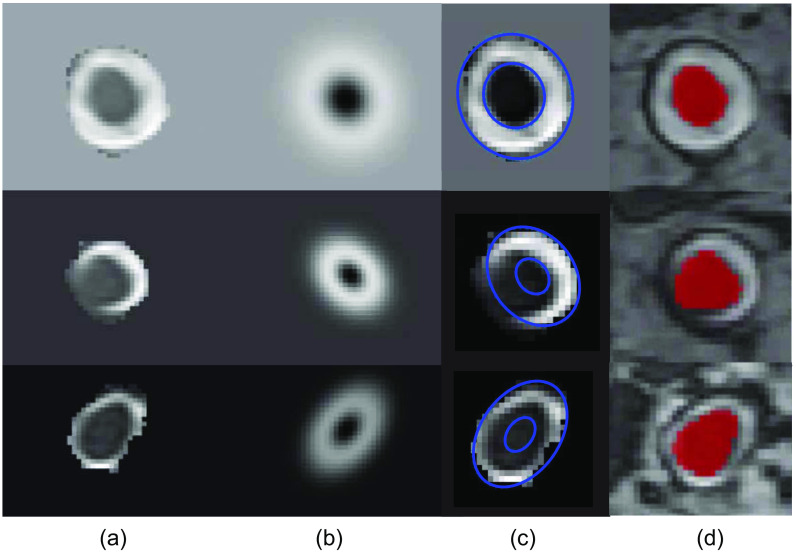
(a) Selected slices of segmented ON and surrounding CSF, ordered from anterior to posterior at the level of the eye (top) to chiasm (bottom). (b) Fitted model of Harrigan et al.^27^ (c) ON and CSF boundaries of the fitted model superimposed on original slice. (d) Segmentation using our proposed segmentation method (in red color).

Figure 10 shows the results of the model fitting procedure to the ON and surrounding CSF, using the method of Harrigan et al.,^27^ in a representative subject. The model fitting accuracy is observed to be higher closer to the eye, where homogeneous CSF surrounds the ON. However, at more distal regions, a systematic bias and erroneous ON diameter can be observed.

**Fig. 11:**
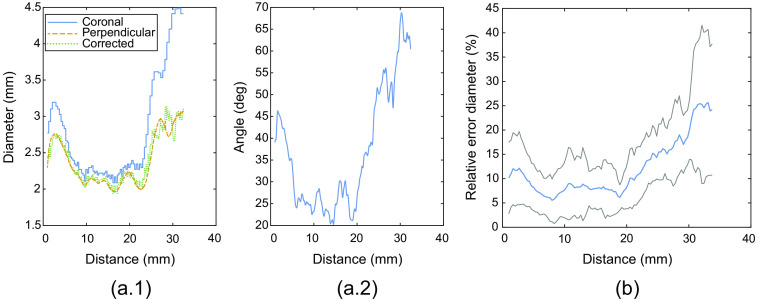
Ablation study. (a) Single subject results: (a.1) Estimated diameter as a function of distance along the centerline, respectively, estimated in the coronal slice (blue line), the perpendicular cross-sectional slice using the proposed method (dashed orange line), and corrected diameter estimated from the coronal slice (dotted green line). (a.2) Angle between centerline and normal line to the coronal plane. (b) Relative error in estimated diameter in the coronal plane relative to the perpendicular cross-section, averaged over all test subjects. The mean error (blue line) with standard deviation (gray lines) over all test subjects is plotted.

The difference in estimated ON diameter when estimated in the coronal plane versus the perpendicular cross-section is shown in Fig. 11. Correcting the coronal diameter with the estimated orientation of the centerline accounts for the observed overestimation. An error of 12±5% is observed across all subjects, with a trend of a larger error toward the periphery of the ON.

## 4 Discussion

In this work, we proposed an automated framework for the segmentation and size quantification of the ON using high-resolution 3D T2-weighted MRI sequences. Our segmentation method achieved segmentations with a high spatial and volumetric agreement to the manual ground truth, while distinguishing the ON from the surrounding CSF, unlike previous studies. In addition, we introduced an automatic quantification method based on centerline extraction to obtain the ON diameter and cross-sectional area along the arc length of the nerve from the eye to the optic chiasm.

### 4.1 Segmentation

Our method achieved outstanding results in ON segmentation, as demonstrated by its high spatial overlap (mean DSC = 0.84) and contour similarity (median HD<0.65  mm and ASD beyond voxel resolution). Comparing our results with other published methods for ON segmentation is challenging since prior studies did not distinguish the ON from the surrounding CSF and employed different datasets with varying imaging sequences. For example, Panda et al.^13^ developed a multiatlas pipeline to segment the eyes, ONs with CSF, and optic chiasm from 3D T2-weighted MR images, reporting a best mean DSC of 0.78 and HD of 2.11 mm for the ONs. Similarly, Mansoor et al.^15^ used an ASM to segment the anterior visual pathway on multisequence MRI data, reporting a mean DSC of 0.79 for the ONs with CSF. Feng et al.^28^ employed a nonautomated gradient edge detection approach to distinguish the ON from CSF on coronal T1-weighted MRI scans, achieving a DSC of 0.81. Deep learning approaches for ON segmentation on MRI have mainly been explored in the context of radiotherapy planning and have generally exhibited low performance for ON segmentation. For instance, Mlynarski et al.^24^ used a 2D U-Net to segment multiple OARs on T1-weighted MR images, obtaining a mean DSC of 0.67 and HD of 6.3 mm for the ONs with CSF. Unlike prior studies that segmented the larger structure of ON including CSF, our method demonstrates improved performance by accurately segmenting the smaller structure of the ON without CSF.

The results presented in Fig. 4 demonstrate that our method performed well in achieving spatial agreement both in regions near the eye, where the presence of CSF provided high contrast with the ON, and in more posterior locations where CSF and other surrounding structures had limited contrast. However, in few cases, our automatic segmentation results were inaccurate due to issues such as disconnected components, voxel misclassifications distant from the ON region, or incomplete segmentations not extending until the optic chiasm. To address these issues, model refinements may be necessary, such as incorporating postprocessing procedures or utilizing a loss function specifically designed for tubular structures to enforce model connectivity.^40^

The evaluation metrics show a high level of consistency between the cross-validation and test-set results, as well as low variance within each set. Our model is thus robust and can effectively handle multicenter input data derived from various MRI scanners and imaging protocols. Since the study involved a clinical dataset of a rare pathology, the amount of annotated data were scarce. By utilizing multicenter data and extensive data augmentation techniques, we managed to increase data variability and still achieve satisfactory and consistent results. To further enhance the model’s robustness and applicability to different ON pathologies, future work should involve expanding and diversifying the dataset, as well as validating the model’s performance in the presence of ON abnormalities.

A substantial increase in DSC was achieved by allowing tolerance of one voxel to the borders. Manually annotating the boundary of the ON, despite being performed with great precision, remains subjective and complicated, particularly in areas with limited contrast to their surroundings. Aside with the effect of interpolating during resampling, this may render noise and uncertain manual ground truths close to the boundaries of the ON. Due to its thin and elongated morphology, ON segmentation is more affected by these issues than other organs. Future research could explore the use of probabilistic ground truth segmentations (i.e., continuous values between 0 and 1)^41^ or adapting the loss function specifically to tubular structure segmentation, such as using a centerline Dice loss.^40^ These approaches could enhance the segmentation accuracy and improve its quantitative evaluation.

### 4.2 Quantification

The developed ON quantification method was able to accurately measure the cross-sectional area and two diameter types, i.e., the diameter calculated from the cross-sectional area (CE diameter) and the MIS diameter. It is important to note that the ON is not a perfectly tubular structure, which can lead to differences between the two types of diameter measurements. Acceptable agreement (ICC>0.7) between manual measurements and automatic measurements was found for all three metrics. In this study, the CE diameter showed the best agreement with the manual diameter measurements, with an ICC of 0.76 and a consistent MAE that was less than the voxel resolution at all three measurement locations.

The Bland–Altman plots reveal a negative mean difference, indicating a tendency for underestimation of ON size. Moreover, there is a small bias toward overestimating smaller ON size and underestimating larger ON size. The observed trend of underestimation may be due to the smoothing process performed by 3D Slicer to generate continuous surface models from raw segmentations. The determination of the offset by the lowest MAE of the origin measurement has likely led to an overestimation of the offset to compensate for the overall underestimation of ON size. This has contributed to the observed bias as smaller ON sizes are generally measured at the origin, where the ON is increasing in size, and larger ON sizes are measured at 3 and 5 mm, where the ON is decreasing in size. Reducing the offset could mitigate the bias, resulting in smaller ON sizes being measured at the origin and larger ON sizes being measured at 3 and 5 mm, but at the cost of increasing the MAE.

Previous studies that aimed at quantifying the ON separately from CSF on MRI scans required manual user input to locate the ON and CSF^28^^,^^42^ or segmentations of the ON with CSF.^27^ The method of Harrigan et al.^27^ was developed on lower resolution data compared to this study (0.27 versus $0.4\;{\mathrm{mm}}^{3}$) and could not widely be applied to our data since it required segmentations of the ON with CSF. Using one representative subject, we visually showed that their model did not perform well on slices where our high-resolution data revealed that the surrounded CSF was limited or not homogeneously distributed around the ON. Moreover, most previous studies extracted ON size based on image intensities in the coronal plane. As we showed in Fig. 11, extracting ON size in the coronal plane results in an overestimation of ON size since the ON is a tortuous structure that bends toward the optic chiasm. In contrast, our developed method based on centerline extraction and measuring ON size perpendicular to its path avoids these limitations and only requires binary segmentations of the ON as input, making it applicable with different segmentation methods and imaging sequences as well.

### 4.3 Limitations and Future Work

The dataset used in this study consisted of MRI scans obtained from retinoblastoma patients. High-resolution 3D T2-weighted MRI scans were available for this pediatric population. However, this high-quality MRI data are not commonly used in clinical practice for orbital or brain imaging. Therefore, it is important to investigate the performance of our segmentation network using clinically available 2D T2-weighted MR images and to extend our analysis to adult populations to enhance its applicability. In addition, since retinoblastoma typically affects young children, our patients were scanned under general anesthesia to avoid motion artifacts. It is important to note that if this method is applied to other ON pathologies in the future, the impact of motion artifacts caused by ocular movement near the globe should be investigated.^12^

In this work, the input size of the segmentation network was adapted to the limited memory capacity of the GPU. Enabling a larger input size could be advantageous, as it allows for the simultaneous segmentation of both ONs and expansion to the entire optic pathway. This would eliminate the need for preprocessing procedures, such as eye centroid detection, alignment, and cropping of the scans. Furthermore, we adhered to a conventional 3D U-Net since attempts to enhance its architecture with additional residual units and attention gating blocks yielded no improvement in segmentation outcomes and required a reduction in the number of filters due to limited GPU memory. Despite the basic nature of our method, it demonstrated high performance and satisfactory segmentations. While more advanced variants of the U-Net could be further explored, previous research has indicated that they may not necessarily result in significant performance improvements and meanwhile increase complexity and require a higher demand on computational resources.^43^

The ON quantification method developed in this study was validated based on manual reference measurements. However, a direct comparison with manual measurements is not straightforward as the offset between the model’s origin and manual reference varies for each nerve, and manual measurements are subjective and sensitive to errors. Moreover, the validation was based on manual measurements limited to three locations near the eye, disregarding the posterior part of the ON. Obtaining accurate manual measurements at multiple points along the entire length of the nerve is challenging and time-consuming, particularly near the posterior part of the nerve due to limited contrast. Nonetheless, validation based on manual references is crucial as manual measurements remain the standard procedure and indicate our model’s ability to realistically quantify ON size.^44^^,^^45^ In future work, we aim to further validate our method and explore its viability based on clinical outcome measures, such as differentiating healthy versus diseased ONs, rather than manual references.

### 4.4 Conclusion

Our study indicates the feasibility of automatic segmentation and quantification of ON volume, diameter, and cross-sectional area on MRI. The proposed framework has the potential for further development toward automated characterization and objective assessment of ON pathology *in vivo*.

## Code Availability

All code used for development of our framework is available at https://github.com/SvElst/Optic_Nerve_Segmentation_3DU-Net.
