# Supplementary material for: Automatic segmentation and quantification of the optic nerve on MRI using a 3D U-Net
Source: J Med Imaging (Bellingham). 2023 May 15;10(3):034501. doi: 10.1117/1.JMI.10.3.034501 (PMC10185127; doi:10.1117/1.JMI.10.3.034501)
Supplement: Supplementary file 1 [file JMI_010_034501_SD001.pdf]

Supplementary Material

Table S1 Comparison of segmentation performances in tenfold cross-validation for various U-Net architectures.

**Metric**	**U-Net**	**U-Net + residual units**[34]	**U-Net + attention gating**[35]
**Spatial (mean ± STD)**			
DSC	0.83 ± 0.04	0.82 ± 0.05	0.82 ± 0.07
Precision	0.85 ± 0.05	0.85 ± 0.06	0.84 ± 0.05
Recall	0.82 ± 0.09	0.81 ± 0.09	0.82 ± 0.11
**Distance (median [IQR])**			
HD95	0.60 [0.42-0.86]	0.67 [0.42–0.85]	0.60 [0.42-0.90]
ASD	0.14 [0.10-0.17]	0.17 [0.11-0.20]	0.14 [0.10-0.19]
**Volumetric**			
ICC	0.88	0.87	0.89

STD=Standard Deviation; IQR=Inter Quartile Range; DSC=Dice Similarity Coefficient; HD95=Hausdorff Distance 95%; ASD=Average Surface Distance; ICC=Intra-class Correlation Coefficient.

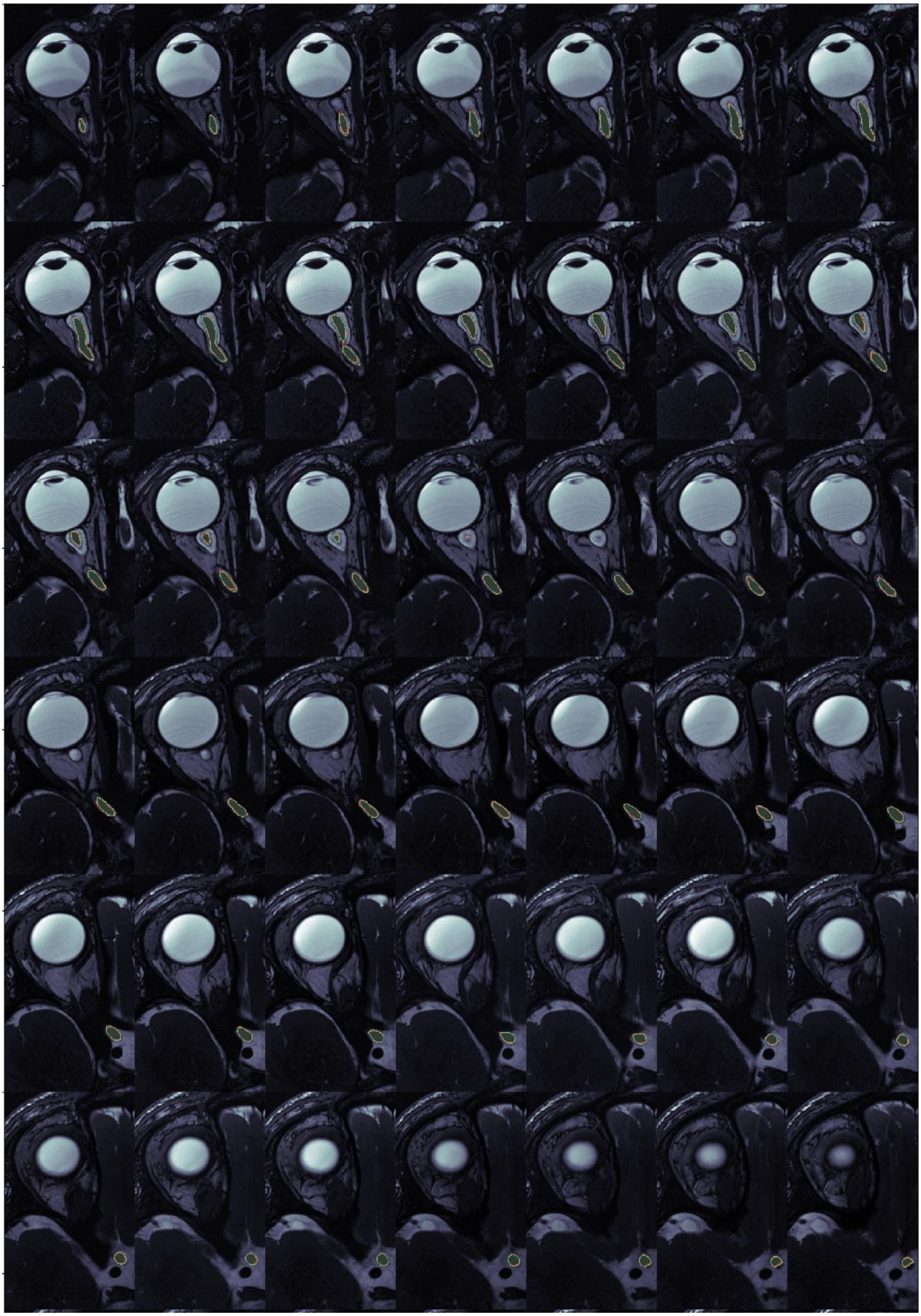

Fig. S1 Segmentation example across all axial slices of one subject (DSC=0.90). Green denotes the segmentation produced by our method, red denotes the manual ground truth.
